# Health Tract, No. 7

**Published:** 1860-02

**Authors:** 


### HEALTH TRACT, No. 7.

### MUSIC HEALTHFUL.

Music, like painting and statuary, refines, and elevates, and ennobles. Song is the language of gladness, and it is the utterance of devotion. But coming lower down, it is physically beneficial; it rouses the circulation, wakes up the bodily energies, and diffuses life and animation around. Does a lazy man ever sing? Does a milk-and-water character ever strike a stirring note? Never. Song is the outlet of mental and physical activity, and increases both by its exercise. No child has completed a religious education who has not been taught to sing the songs of Zion. No part of our religious worship is sweeter than this. In David's day it was a practice and a study.

### YOUNG OLD PEOPLE.

Some look old at less than forty; others beyond threescore have the vivacity, the sprightliness, and the spring of youth. One of the most active politicians of the times is now in his seventy-fifth year, and yet goes by the name of "the ever youthful Palmerston," and with the weight of nations on his shoulders, will find time to take a rapid ride on horseback daily, from ten to twenty miles. "The heavy cares and severe labors of the Earl of Malmesbury average eleven hours a day," and yet at the age of "fifty years, he is scarcely above forty in appearance." It is by no means an uncommon thing to read the deaths of men and women of the English nobility at eighty and ninety years, to be accounted for in part by their taking time to do things, and thereby doubling the time for doing them. The British are a dignified people, manly, mature; a deliberative people, with the result of being as a nation, the most solid, the most substantial, and the greatest on the globe. They are worthy of that greatness, and we above all the peoples should be proud of it. Americans, on the other hand, are a hasty race; their habitual hurries and anxieties eat out the very essence of life before half that life is done, and all bloodless, fidgety, skinny, and thin, we are but "a vapor that appeareth for a little time, and then vanisheth away."

